# Clinicopathological features and prevalence of prostate cancer in Aseer, Saudi Arabia

**DOI:** 10.15537/smj.2022.43.7.20210758

**Published:** 2022-07

**Authors:** Hassan M. Otifi, Osama M. S. Abdul-Wahab, Mishari H. Al-Shyarba, Majed S. S. Al Fayi, Abdullah H. Al Murea, Elhem Yacoub

**Affiliations:** *From the Department of Pathology (Otifi); from the Department of Surgery (Al-Shyarba), College of Medicine; from the Department of Clinical Laboratory Sciences (Abdul-Wahab, Al Fayi), Faculty of Applied Medical Sciences, King Khalid University, from the Department of Laboratory (Al Murea), Aseer Central Hospital, Abha, Kingdom of Saudi Arabia, and from the Specialized Unit of Mycoplasmas, Laboratory of Molecular Microbiology, Vaccinology, and Biotechnology Development (Yacoub), Institut Pasteur de Tunis, University of Tunis El-Manar, Tunis, Tunisia.*

**Keywords:** prostate cancer, incidence, Aseer region, Saudi Arabia

## Abstract

**Objectives:**

To determine the prevalence and characterize prostate cancer (PC) cases in Aseer, Saudi Arabia.

**Methods:**

This study involved 883 patients who consulted physicians in Aseer Central Hospital, Abha, Saudi Arabia, for prostate issues between the years 2008-2018. All patients underwent digital rectal examination and measurement of their serum prostate-specific antigen levels. For patients who presented abnormal digital rectal examination findings and elevated prostate-specific antigen levels, prostate biopsies were recommended. Specimens were histopathologically examined to differentiate between malignant and benign tumors.

**Results:**

Among the 883 included patients, 132 (15%) underwent a prostate biopsy and were found to have a tumor. Histopathological examination confirmed malignancy (PC) in 77 (8.7%) patients. The absolute majority of the patients diagnosed with PC (96%) were aged >60 years and almost all of them (92%) were found to have a high prostate-specific antigen level of >4 ng/ml.

**Conclusion:**

Prostate cancer appears to be a serious disease in Aseer, Saudi Arabia. Further studies aimed at determining the causes of this type of cancer and understanding its mechanisms are warranted.

**P**rostate cancer (PC) is one of the most common cancers that affect men worldwide.^1^ At early stages, PC can grow slowly and cause no symptoms or troubles, in which case minimal treatment would be enough to cure it.^1^ However in advanced stages, more-serious complications such as urinary hesitancy, frequent urination, or urinary retention may be observed.^2^ Compared with other cancer types, the etiology of PC is largely unknown. Many risk factors have been linked to PC, of which age, genetic factors, and ethnicity are the most suggested.^3^ In addition, several other factors, including diet, obesity, infections, and inflammation were also evoked.^4^ Abnormal digital rectal examination (DRE) and elevated plasmatic levels of prostate-specific antigen (PSA) are the most common findings that initiate further investigation for malignancy in the prostate gland. Owing to its effectiveness, tissue biopsy is still the standard of care for confirming and validating the presence of cancer.^1^

The incidence rate of this type of cancer varies across regions and populations. In the Kingdom of Saudi Arabia (KSA), almost 700 new cases of PC and >200 deaths were reported in the latest GLOBOCAN report published in December 2020.^5^ In terms of incidence, PC ranked 12 among the 35 cancer types found to affect the Saudi population. With regard to mortality, this cancer type ranked 19^th^ among the most common causes of cancer-related death in men.^5^ Apart from the annual international reports published by the World Health Organization (WHO), a few local studies have investigated PC, although the epidemiology of this cancer remains underestimated in KSA and the statistics are not fully updated.^6-9^

In this study, we aimed to report new data on the incidence of PC during the last 10 years in a group of Saudi patients from Aseer region (southwest of KSA).

## Methods

This retrospective study consisted of 883 patients with different issues in the prostate gland (such as, enlargement, pain, hard mass, induration, and asymmetry) who were referred to Aseer Central Hospital, Abha, KSA, during a period of 10 years (from 2008 to 2018). All the patients were physically examined with DRE and their serum PSA levels were measured. Inclusion criteria included patients’ medical conditions, diagnosed histopathologically confirmed prostatic adenocarcinoma, tumor stage, PSA level, presence of Gleason grade pattern, control cases (benign prostatic hyperplasia [BPH]), and signed informed consent. The exclusion criteria included any medical condition that might interfere with the evaluation of the study objectives; patients with contraindications for either prostatectomy or radiotherapy to the prostate.

This study was approved by the Internal Review Board committee of Aseer Central Hospital, Academic Affairs for Training and Research, Abha, KSA (approval No.: 26-12-2017). All the enrolled patients have signed an informed consent.

**Figure 1:**
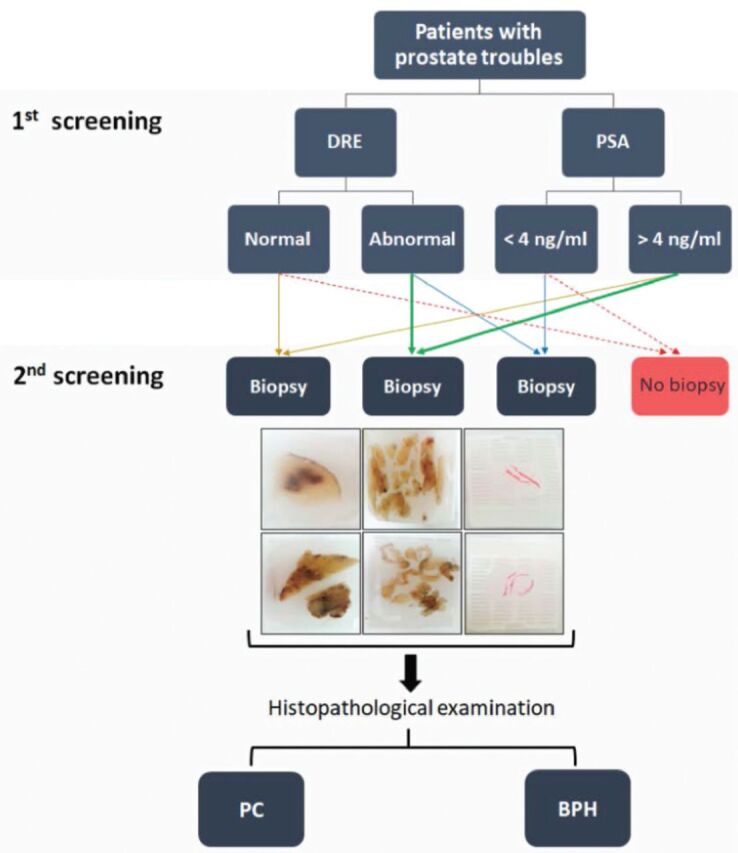
- Flowchart of the study design, showing the different screening steps for specimen sorting. On the basis of DRE findings and PSA levels, biopsy was either highly recommended (bold solid green arrows) or moderately recommended (solid blue and yellow arrows) or not recommended at all (red dashed arrows). DRE: digital rectal examination, PSA: prostate-specific antigen, PC: prostate cancer, BPH: benign prostatic hyperplasia.

For the patients who presented suspicious DRE findings or elevated PSA levels, prostate biopsy was recommended. On a case-by-case basis, some specimens were obtained from the patients using transrectal ultrasonography-guided biopsy; and others, by a surgical procedure for transurethral resection of the prostate. Malignant prostate tumors (PC) were differentiated from benign tumors (BPH) after a scrupulous histopathological examination under light microscopy was carried out by anatomic pathologists. A flow diagram of the patient enrollment is presented in Figure 1. Details on the patients age were collected from their medical records.

### Statistical analysis

A basic statistical analysis with Microsoft Excel software was carried out to schematically present prevalence results of the PC disease in the Saudi men population studied in this paper.

## Results

**Figure 2:**
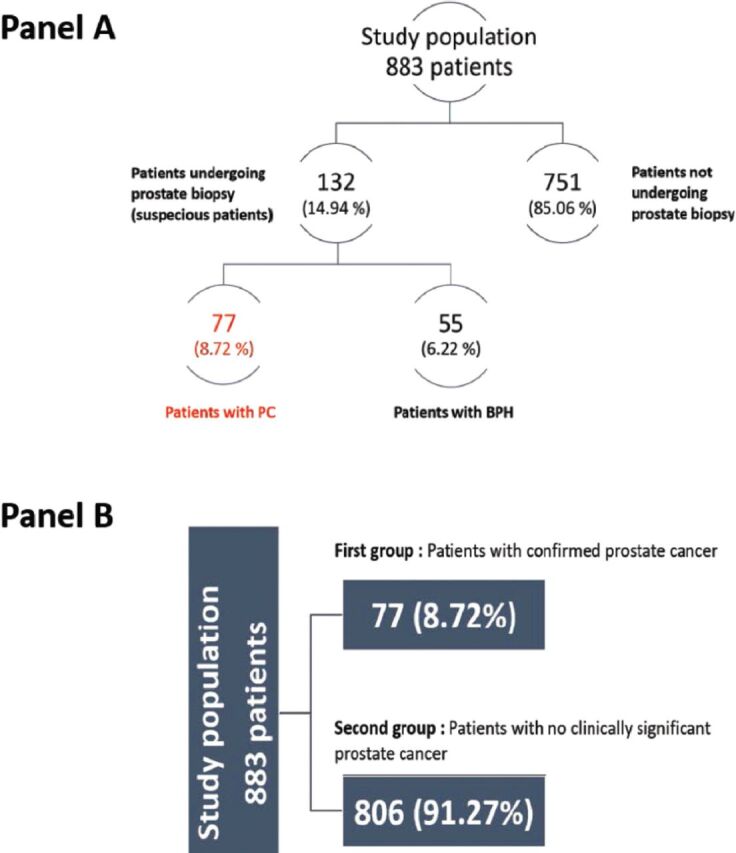
- Prevalence of prostate cancer in the Saudi population from Aseer province included in the study. Panel A) partition of the enrolled patients according to prostate biopsy screening and histopathological examination. Panel B) division of the whole study population based on PC diagnosis. PC: prostate cancer, BPH: benign prostatic hyperplasia

After DRE and PSA measurement, 132 of the 883 patients included in this study (approximately 15%) were suspected as having a prostate tumor and thus underwent biopsy. On the basis of the histopathological examination results of the biopsies collected from the patients with a suspected prostate tumor, malignant tumors were attributed to 77 patients. The remaining men were diagnosed with BPH. Considering the total number of patients included in this study, the prevalence of PC in Aseer region during the decade from 2008 to 2018 was 8.7%, whereas the prevalence of BPH was 6.2% among the patients who underwent prostate biopsy (for a suspected prostate tumor). These data are schematically summarized in Figure 2.

From a more conclusive point of view, the 883 patients enrolled in this study were divided into 2 groups, one including 77 (8.7%) patients who were effectively diagnosed with PC on the basis of their histopathological examination results and the other consisting of the remaining 806 (91.3%) patients. In contrast to the patients in the first group, those in the second group were broadly considered as subjects with BPH because the signs of the clinical manifestations were either absent or not strong enough to confirm the serious diagnosis of significant PC (Figure 2).

**Figure 3:**
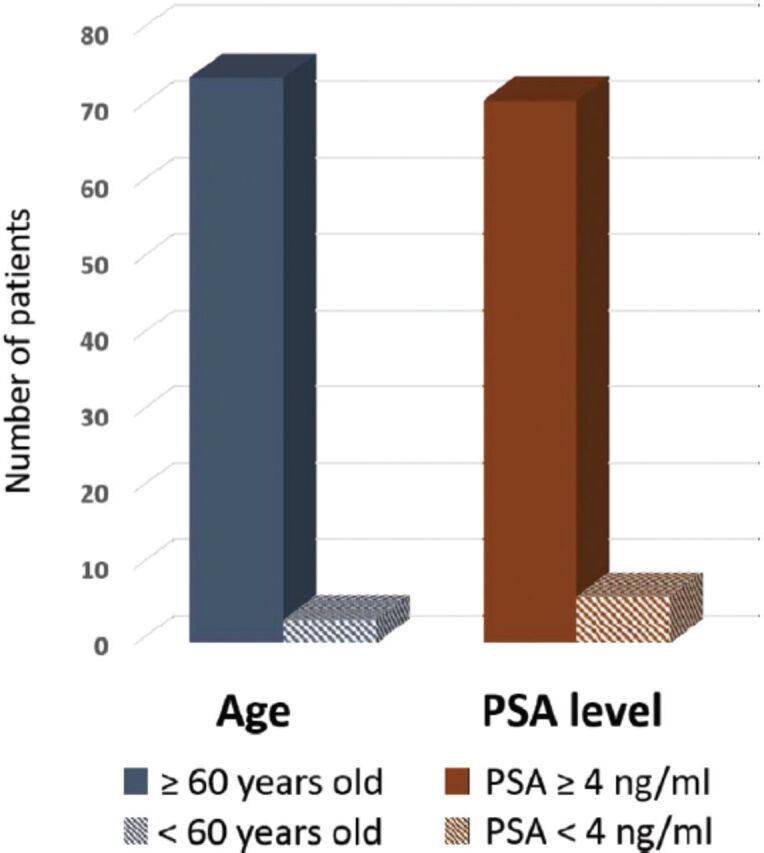
- Grouping of the patients with prostate cancer according to age and blood prostate-specific antigen (PSA) level.

According to age and PSA level, similar distributions of the 77 patients diagnosed with PC were observed. Most patients (96%) were aged >60 years, and only 3 (4%) men were aged <60 years. Likewise, a clear majority of these patients with cancer (92%) were found to have high PSA levels (>4 ng/ml). The remaining 6 (8%) men showed normal PSA concentrations in their blood. The distributions of the patients with PC according to age and PSA level are presented in Figure 3.

## Discussion

This study was carried out to estimate the prevalence of PC in Aseer region and to characterize the affected male population. Therefore, we included patients with prostate diseases who consulted the regional hospital. According to our results, the prevalence of PC among the Saudi patients from Aseer between 2008-2018 was 8.7%. Information on PC in South KSA is rare. We presumed that no data were available regarding the occurrence of this type of cancer in the region of Aseer. In fact, apart from a brief notification reported in an old study published 30 years ago, which showed that the prostate is among the most common cancer sites in males in Aseer province, no other study has particularly outlined the occurrence or patterns of PC cases in this region.^10^ Thus, no comparison could be made to assess and monitor the statistics of PC over time in this Saudi province. Nevertheless, a few studies reported data regarding the incidence rates of PC in other regions of KSA. In a retrospective cohort study carried out in Riyadh, 31 (2%) of 1521 Saudi patients were found to have an adenocarcinoma.^6^ In another similar study in Jeddah, a more significant incidence of PC was reported (28.5%).^8^

Moreover, almost all patients diagnosed with PC (96%) were aged >60 years. This may reflect the possible correlation between patient’s age and the incidence of PC. The older the patient was, the higher was his risk of developing PC. This observation was reported before. Prostate cancer is considered one of the prostate diseases that present serious health challenges in elderly men.^11^ A previous study showed that clinically significant PC generally affects men in their 60’s (median age is 66 years) and that PC cases in men under the age of 50 years were rare.^11^ However, the possibility of PC onset years before the 6^th^ decade of life was suggested. Owing to these paradoxical observations, the primary cause that triggers prostate carcinogenesis is difficult to establish, especially that besides age, many other possible causative factors could be involved in male genitourinary malignancies.^4^ Moreover, the increasing number of men diagnosed with PC is believed to be a result of the practice of PSA screening. According to the Unied States of America’s National Cancer Institute, men with blood PSA levels of >4 ng/ml are recommended to undergo prostate biopsy for examination of prostatic tissue for the presence of abnormal tumor cells.^12^ In the Saudi population included in this study, we found that 92% of the patients with PC had recorded PSA levels of >4 ng/ml. The commonly used serum PSA test is greatly useful in the early diagnosis of PC. However, the test may sometimes lead to many unnecessary biopsies, overdiagnosis of PC, and thus overtreatment. The PSA test is highly sensitive but not exclusively specific to PC, especially within the gray zone (PSA values between 4-10 ng/ml). This is explained by the fact that even in patients with BPH or another form of prostatitis, high PSA levels could be observed. Therefore, PSA level was judged to be a good but not perfect PC biomarker.^13^

### Study strengths and limitations

This paper presents an update on the situation in Aseer province in KSA regarding the prevalence of the serious disease of PC. This subject was not studied for years, a fact that emphasizes the usefulness of the presented results. With a prevalence rate of approximately 9%, we clearly report the considerable spread of PC in Aseer region between the years 2008-2018. Although, 10 years to study cancer occurrence in a local population (with relatively low density) could be a short period to correctly assess the spread of this chronic and slow-developing disease.

In conclusion, clinical picture of PC is complex and its diagnosis is not easy. Healthcare providers should pay too much attention before announcing the diagnosis since many factors, mainly PSA screening, could have an important impact. Besides our present study, other local studies have also monitored the epidemiological status of PC in different regions in the KSA. More attention and prompt screening are needed to determine the causes and understand the mechanisms of this type of cancer.
